# Globulol from *Alpinia oxyphylla* Miq. Enhances the pharmacological effects of anti-PD-1 drugs in combination by reducing PD-L1 expression in hepatocellular carcinoma

**DOI:** 10.3389/fphar.2026.1728692

**Published:** 2026-02-20

**Authors:** Zhe Wang, Yinghong Zhong, Jicheng Hu, Peishi Xie, Yuan Zhao, Cunzhen Jiang, Lu Lu, Mingyan Zhou, Jian Xu

**Affiliations:** 1 Hepatobiliary and Liver Transplantation Department of Hainan Digestive Disease Center, The Second Affiliated Hospital of Hainan Medical University, Haikou, Hainan, China; 2 Department of Hepatobiliary and Pancreatic Surgery, The Second Affiliated Hospital of Hainan Medical University, Haikou, Hainan, China; 3 Institute of Clinical Medicine, The Second Affiliated Hospital of Hainan Medical University, Haikou, Hainan, China; 4 Key Laboratory of Emergency and Trauma of Ministry of Education, The First Affiliated Hospital of Hainan Medical University, Haikou, Hainan, China

**Keywords:** Alpinia oxyphylla Miq., anti-PD-1 drugs, globulol, hepatocellular carcinoma, PD-L1, tislelizumab

## Abstract

**Introduction:**

Globulol is a terpenoid metabolite isolated from *Alpinia oxyphylla* Miq. Previous studies have demonstrated that terpenoid metabolites exhibit anti-inflammatory, antioxidant, and antitumor activities. However, the exact mechanisms by which Globulol impacts HCC remain obscure.

**Materials and Methods:**

The influence of Globulol on HCC cell lines was assessed *in vitro* employing the CCK8, EdU cell proliferation assays, a three-dimensional tumor spheroid model, and flow cytometry. The combination effects of Globulol and anti-PD-1 was explored in an HCC and CD8^+^T cell co-culture model. The molecular pathways influenced by Globulol in HCC were delineated through transcriptomic sequencing, molecular docking, bioinformatics approach, cell transfection, quantitative reverse transcription-PCR, and Western blot analysis.

**Results:**

Globulol notably decreased the viability of Huh1/Huh7 cells in a concentration-dependent manner. It was found to prompt the secretion of the chemokine CCL4 by tumor cells, enhancing the infiltration of cytotoxic CD8^+^T cells into the HCC microenvironment. Further research has indicated that globulol inhibits PD-L1 expression by targeting the PAK4-pAKT-STAT3 axis, improves the immunosuppressive microenvironment of HCC, and enhances the pharmacological effects in combination with Tislelizumab.

**Conclusion:**

These results suggest that Globulol can promote a microenvironment permissive to PD-1 blockade, thereby exerting anti-HCC activity. Hence, the combination of Globulol and PD-1 blockade may be a promising strategy for HCC treatment.

## Graphical Abstract

**Figure:**
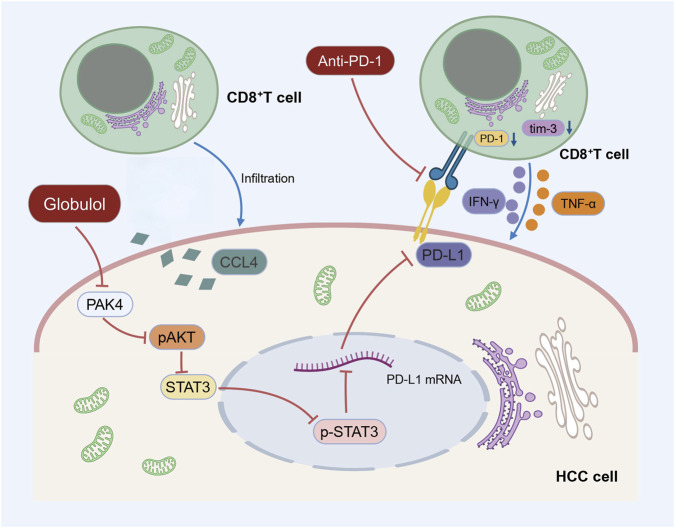

## 1 Introduction

Hepatocellular carcinoma (HCC), a malignant growth originating from the liver’s epithelial or mesenchymal tissues, is strongly associated with risk factors such as chronic hepatitis, viral infections, obesity, and alcohol consumption (Chidambaranathan-Reghupaty et al., 2021). Representing a significant global health challenge, HCC is estimated to affect over 1 million individuals annually by 2025 (Galle et al., 2018). It is the sixth most common cancer worldwide, holds the third-highest mortality rate globally, and has a five-year survival rate ranging from 5% to 30% (Bray et al., 2024). Available treatments for HCC encompass targeted therapy, interventional embolization, surgery, radiotherapy, and chemotherapy. For early-stage HCC, surgical resection and liver transplantation are the most effective therapies. Nevertheless, the low rate of early diagnosis and the rapid progression of the disease often limit the pharmacological effects of traditional treatments in advanced-stage liver cancer (LC) (Vogel et al., 2025).

*Alpinia oxyphylla* Miq., a classic botanical drug with a centuries-old history of medicinal and dietary use in China, holds prominent ethnopharmacological significance (Zhao et al., 2016). Revered as one of the “Four Famous South Medicines,” its dried fruits (*Alpinae Oxyphyllae* Fructus) have been extensively documented in classical Chinese medical texts. Ben Cao Gang Mu (Compendium of Materia Medica) recorded its effects of “nourishing the kidney, consolidating essence, and regulating qi” (Park et al., 2022), while Shi Liao Ben Cao (Dietary Materia Medica) noted its potential in tonifying the spleen and soothing the nerves (Qiu et al., 2023). Globulol, a major sesquiterpene terpenoid isolated from *Alpinia oxyphylla* Miq., is considered one of its key active constituents. Modern pharmacological studies have demonstrated that globulol possesses diverse pharmacological activities. Notably, its ethnopharmacological application in antitumor therapy has been well-documented in relevant literature (Yang and Guo, 2007).

Sesquiterpenes, a subclass of terpenoids, feature a 15-carbon isoprenoid backbone that supports their anticancer activity, with structural subtypes like globulol’s guaianolide form enabling binding to cancer-related targets. Key anticancer mechanisms include modulating oncogenic pathways (PI3K/AKT/mTOR, STAT3, MAPK/ERK), inducing ROS-mediated apoptosis, and inducing autophagy and ferroptosis (Xu et al., 2025). While our prior study using liquid chromatography-mass spectrometry detected the terpenoid Globulol in the *Alpinia oxyphylla* Miq. extract, its anti-HCC pharmacological effects and underlying molecular mechanisms have not yet been fully determined.

The programmed cell death protein-1 (PD-1) and its ligand (PD-L1) are recognized as negative regulators of immune function, and their role in promoting tumor immune escape has attracted considerable interest. Tumor cells expressing high levles of PD-L1 can evade immune surveillance. The binding of PD-L1 to PD-1 on T cells suppresses T cell activity and disrupts the cancer-immune cycle (Dermani et al., 2019). In LC tissues, CD8^+^T cells and Kupffer cells show elevated expression of PD-1 and PD-L1, respectively. This interaction impairs the function of effector T cells in HCC, leading to reduced secretion of anti-tumor cytokines and cytotoxic granules, and ulitimately fostering an immunosuppressive tumor microenvironment (TME) (Wu et al., 2009). Based on the significant success observed in clinical trials, ten α-PD-1 and three α-PD-L1 antibodies have been approved for the treatment of various cancers types. Nevertheless, the low response rate of α-PD-1/PD-L1 therapy remains to be resolved (Yi et al., 2022). However, the pharmacological activity and molecular mechanisms by which Globulol from *Alpinia oxyphylla* Miq., in combination with anti-PD-1 agents, modulates the immune microenvironment and downregulates PD-L1 expression in HCC have yet to be fully elucidated.

In the present study, we employed methods including RNA-sequencing (RNA-seq) and three-dimensional (3D) tumor sphere culture to evaluate the potential of Globulol in exerting combined pharmacological effects with anti-PD-1 drugs for HCC treatment, with the aim of providing a scientific basis for its future clinical application.

## 2 Materials and methods

### 2.1 Antibodies and reagents

Globulol (PubChem CID: 12304985, Catalog Number: B29181, Purity: ≥ 98%) was supplied by Yuanye Biotechnology (Shanghai, China). Tislelizumab (TIS, BGB-A317) was supplied by BeiGene, Ltd. (Beijing, China). The cell lines used in this study were obtained from Wuhan Oyster Mushroom Biotechnology Co., Ltd., Bena Bioengineering Technology Research Center, and Wuhan Huofai Biotechnology Co., Ltd. The fetal bovine serum and Dulbecco’s Modified Eagle Medium (DMEM) were supplied by Gibco. Apexbio (K1018) provided the CCK8 reagent. Beyotime Biotechnology provided the EdU cell proliferation kit (C0075S), cell cycle analysis kit (C1052), CFDA-SE Cell Proliferation Detection Kit (C0051), LDH assay kit (C0016), PAK4 inhibitor (PF-03758309), Human CD8^+^T Cells Positive Selection Kit (071A103.11), and the apoptosis assay kit (40302ES50). IPHASE Co., Ltd, BeiGene, Ltd., and Yeasen Biotechnology (Shanghai) Co., Ltd also supplied various reagents. Abcam and Proteintech provided the antibodies used in this study. The antibodies against PAK4 (ab314765), AKT (60203-2-Ig), phospho-AKT (80455-1-RR), STAT3 (ab68153), phospho-STAT3 (ab76315), PD-L1 (28076-1-AP), and GAPDH (60004-1-Ig) were acquired. The Guangzhou Tianyi Huiyuan Biotechnology Co., Ltd. offered primers for genes such as GAPDH, CCL2, CCL3, CCL4, CCL5, CXCL9, CXCL10, IFN-γ, and TNF-α.

### 2.2 Cell lines and culture conditions

HCC cell lines Huh1 and Huh7, and the human hepatic stellate cell line LX-2 were purchased from Wuhan Procell Life Science and Technology Co., Ltd. (Wuhan, China). Primary human CD8^+^ T cells were obtained from Xiamen Immocell Biotechnology Co., Ltd. (Xiamen, China). All cell lines were authenticated by short tandem repeat (STR) profiling to confirm their identity, and no cross-contamination was detected in subsequent culture processes. Huh1, Huh7, and LX-2 cells were cultured in Dulbecco’s Modified Eagle Medium (DMEM) high-glucose medium (Gibco) supplemented with 10% fetal bovine serum (FBS, Gibco, United States), 100 mg/mL penicillin, and 100 mg/mL streptomycin. Primary human CD8^+^T cells were maintained in RPMI-1640 medium (Gibco) containing 10% FBS, 100 mg/mL penicillin, and 100 mg/mL streptomycin. All cells were incubated in a humidified cell culture incubator at 37 °C with 5% CO_2_. Prior to co-culture experiments with HCC cells, CD8^+^T cells were pre-activated to enhance their functional activity: CD8^+^T cells were stimulated with ImmunoCult™ CD3/CD28/CD2 T Cell Activator (STEMCELL Technologies) and IL-2 for 48 h at 37 °C with 5% CO_2_.

### 2.3 Cell viability assay

In 96-well plates, cells were seeded at 3,000 cells per well and treated with varying doses of Globulol (0–320 μM) for a duration of 24 h. Post-treatment, the supernatant was removed, and each well received 100 μL of medium supplemented with 10% CCK8. Absorbance was measured at 450 nm using a spectrophotometer to assess cell viability.

### 2.4 EdU assays

The cells were then incubated with a 2x EdU solution for 2 hours after being treated with several doses of Globulol. After that, each well was fixed for 15 min with 4% paraformaldehyde. Permeation of the cells was carried out for a further 15 minutes using 0.25% Triton X-100 following fixation. Before the last staining stage with Hoechst for 10 min, cells were incubated with a click additive solution for 30 min. Afterwards, they were observed under a fluorescence microscope.

### 2.5 Flow cytometry (FC)

Huh1 and Huh7 cells were treated with different concentrations of Globulol for 24 h, followed by resuspension in 1× binding buffer. The cells were stained with Annexin V-FITC and PI according to the directions of the apoptosis kit, and then left to incubate in darkness for 10–15 min. Afterwards, Fc was used for quantitative analysis of apoptotic rates. Cells were fixed with 80% ethanol, stained with PI, and cultured in darkness at 37 °C for 30 min prior to Fc evaluation in order to conduct cell cycle study. CFDA-SE was integrated into the co-culture system of 6-well plates treated with different doses of Globulol and TIS. CD8^+^T cells, post co-culture, were separated using the Human CD8^+^T Cells Positive Selection Kit protocol, and proliferation metrics were evaluated via FC.

### 2.6 Viability assay for live/dead cells

Forty microliters of Cytoport Biotech’s Biomimetic Matrix Gel for three-dimensional (3D) Cell Culture (CulX I) and 50 μL of Huh1 and Huh7 cell suspensions were spread out in every well of a 96-well plate. One hundred microliters of culture media was added once the gelling process had culminated. The cells were cultured for 6 days, then stained with calcein-AM/PI, subjected to Globulol for 24 h, and finally evaluated under a fluorescence microscope.

### 2.7 3D migration assay

A 50 μL suspension of Huh1 and Huh7 cells mixed with Biomimetic Matrix Gel was placed into each well of a 96-well plate. After solidification, 100 μL of medium was added and cells were cultured for 6 days. Post transfer to a 6-well plate, tumor spheroids were exposed to varied concentrations of Globulol for 12 h, stained with Calcein-AM, and visualized with a fluorescence microscope.

### 2.8 Co-culture transwell model

In 12-well plates equipped with Transwell inserts, 80 μL of Biomimetic Matrix Gel was applied to the bottom of the upper chamber. After solidification, CD8^+^T cells were seeded into the upper chamber at a density of 1 × 10^5^ cells/well, and stimulated with ImmunoCult™ CD3/CD28/CD2 T Cell Activator (STEMCELL Technologies) and IL-2 for 48 h. The stimulation was performed in RPMI-1640 medium (Hyclone) supplemented with 10% fetal bovine serum (FBS, Gibco) under the condition of 37 °C and 5% CO_2_. Subsequently, hepatocellular carcinoma (HCC) cells (Huh1 and Huh7) were added to the lower chamber of the 12-well plate at a ratio of 25:1 (relative to CD8^+^T cells, approximately 4,000 cells/well) and cultured in serum-free Dulbecco’s Modified Eagle Medium (DMEM). At the initiation of co-culture, Globulol (80 μM) and TIS (57.14 mg/L) were added to the lower chamber (Figure 5A). Subsequently, the activated CD8^+^T cells and HCC cells were co-cultured in a humidified incubator at 37.0 °C with 5% CO_2_ for 24 h. After the 24 h co-culture period, the supernatant and floating CD8^+^T cells were removed, and the biomimetic matrix was wiped off. Cells in the upper chamber were stained with Calcein-AM and imaged using a fluorescence microscope.

### 2.9 3D co-culture model

Using previously described techniques, a 3D tumor spheroid model comprising Huh7 cells was constructed. These spheroids were transferred to a 24-well plate, and CD8^+^T cells were seeded into the plate at a density of 1 × 10^4^ cells per well in RPMI-1640 medium containing 10% FBS. Subsequently, the co-culture system was treated with Globulol (80 μM) and TIS (57.14 mg/L), and maintained for 24 h. After the incubation period, the 3D tumor spheroids were observed under a bright field microscope.

### 2.10 LDH assay

In a 24-well format, CD8^+^T cells and Huh7 cells were co-cultured and then treated with various Globulol and TIS combinations for 24 h. The supernatant was subsequently collected for LDH activity measurement using a microplate reader.

### 2.11 RNA-seq and data analysis

RNA-seq was performed on Huh7 cells, both with and without drug treatment. Following extraction, total RNA was assessed for its purity, concentration, and integrity. The Illumina HiSeq platform’s paired-end sequencing technique was utilized for comprehensive sequencing of the samples. Differentially expressed genes (DEGs) were identified using the DESeq2 software package. The selection criteria for DEGs were defined as absolute log_2_ fold change (|log_2_FC|) ≥ 1 and a statistical significance of P value ≤0.05. Analyses were carried out utilizing the Kyoto Encyclopedia of Genes and Genomes (KEGG) database and the TopGO database, respectively, to implement gene ontology (GO) functions. The analysis’s visual enrichment plots were also made easier to create with the use of gene set enrichment analysis (GSEA) software.

### 2.12 Cell transfection

The sequences for PAK4 overexpression and knockdown were acquired from Shanghai Jikai Gene Chemical Technology. Huh7 cells were transfected with these plasmids using PEI 40K Transfection Reagent (Servicebio, Wuhan, China) following the manufacturer’s guidelines. Post-transfection, cells were exposed to Globulol for 24 h. Subsequently, gene and protein samples from these cells were harvested for further analysis.

### 2.13 Quantitative reverse transcription-PCR (qRT-PCR) analysis

The co-culture approach was used to isolate CD8^+^T cells by employing a Human CD8^+^T cell Positive Selection Kit. To prepare for qPCR, total RNA was extracted using the Eastep® Super Total RNA Extraction Kit and then converted to cDNA using Hifair 1/3 1st Strand cDNA Synthesis SuperMix. Using a thermal cycler, the qRT-PCR was carried out with SYBR Green. Table 1 lists the primers that were used. The 2^−ΔΔCt^ technique was used to process the data after normalizing the expression levels of particular genes to GAPDH.

**TABLE 1:** Primer sequences for qRT-PCR.

Gene	Forward (5′–3′)	Reverse (5′–3′)
CCL2	ATG​AAA​GTC​TCT​GCC​GCC​CTT	TGA​AGA​TCA​CAG​CTT​CTT​TGG​G
CCL3	CCT​GCT​GCT​TCT​CCT​ACA​GC	TCA​GGC​ATT​CAG​TTC​CAG​GTC
CCL4	ATG​AAG​CTC​TGC​GTG​AGG​GC	CTG​GGT​CTG​GAG​CCA​GTT​TG
CCL5	CCT​GCT​GCT​TTG​CCT​ACA​TT	ACA​CAC​TTG​GCG​GTT​CTT​TC
CXCL9	TGC​AGG​ACT​CTA​AGG​CGG​AA	CAG​GTG​GAC​CAT​CTG​GTT​CG
CXCL10	GGT​GAG​AAG​AGA​TGT​CTG​AAT​C	GTC​CAT​CCT​TGG​AAG​CAC​TGC​A
IFN-γ	TGG​AGA​CCA​TCA​AGG​AAG​ACA​G	TGC​TTT​GCG​TTG​GAC​ATT​CAA​G
TNF-α	CAG​AGG​GAA​GAG​TTC​CCC​AG	CCT​TGG​TCT​GGT​AGG​AGA​CG
PD-1	CTG​GCG​TTG​ACA​GAC​TGG​TA	TGC​TGG​GAC​GAT​CTT​CAC​TG
TIM-3	GCT​GCA​CCT​GGT​GAA​GAA​CT	AGG​CAG​GGT​CTG​GTA​GTT​CC
PD-L1	TGC​GGA​CTA​CAA​GCG​AAT​CA	CTG​CTT​GTC​CAG​ATG​ACT​TCG​G
GAPDH	CCTTCCGTGTCCCCACT	GCCTGCTTCACCACCTTC

### 2.14 Molecular docking

SDF files of Globulol (CAS number: 489–41-8) were retrieved from the Pubchem database. Structural details of pertinent target proteins were obtained from the PDB database. These protein structures were refined using Pymol-2.1.0 software, followed by hydrogen addition and charge settings using AutoDockTools-1.5.6. Molecular docking was executed with the vina-2.0 module within the pyrx software suite, calculating the binding energies. Visual representations were analyzed using Pymol and the Discovery Studio 2019 Client tools.

### 2.15 Western blot (WB) analysis

A RIPA buffer with 1% protease inhibitor, purchased from Beyotime Biotechnology Co., Ltd., was used to break down the cells. Afterwards, the protein concentration was measured using the supernatant. After the protein samples were prepared, they were transferred to PVDF membranes and separated using SDS-PAGE. After 1 hour of blocking with 5% skim milk, the membranes were incubated with primary antibodies at 4 °C throughout the night. After three washes with 1% TBST the next day, the membranes were incubated with secondary antibodies conjugated with HRP for 1 hour. Protein bands were seen using a Tanon imager and band intensity was assessed using ImageJ software after three more washes.

### 2.16 Statistical analysis

At least three independent experiments were conducted for each experimental condition. The mean ± standard deviation is the result of data analysis performed using GraphPad Prism 8 program. A two-tailed paired Student’s t-test, with a significance level of *P* < 0.05, was used to conduct comparative analyses between the two datasets.

## 3 Results

### 3.1 Globulol reduces viability and proliferation of HCC cells

**FIGURE 1:**
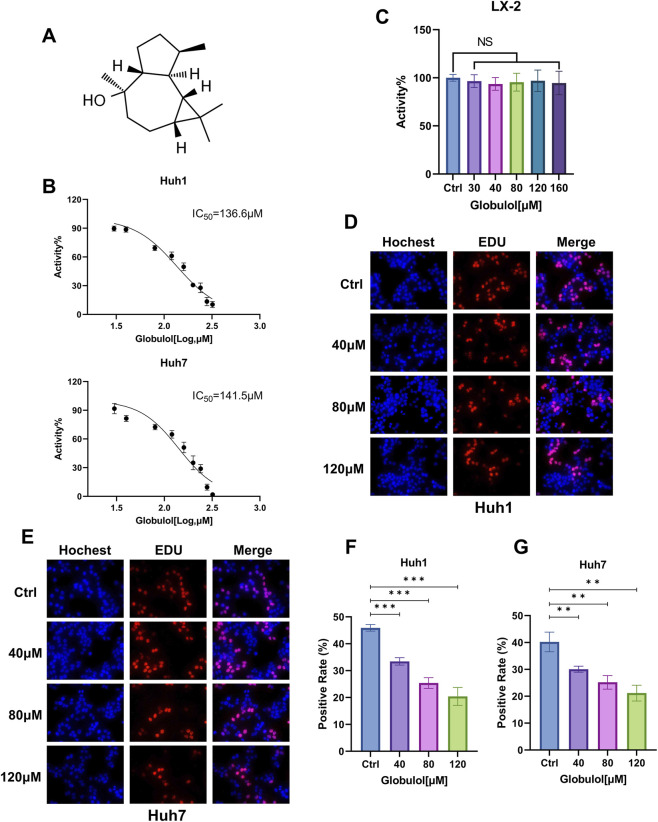
Globulol leads to a reduction in proliferation and enhances cytotoxic effects in Huh1 and Huh7 cell lines. **(A)** The chemical structure of Globulol. **(B)** The viability of Huh1 and Huh7 cells subjected to Globulol treatment, and the IC50 values were determined. **(C)** The viability of LX-2 cells subjected to treatment with Globulol. The proliferation of Huh1 **(D,F)** and Huh7 **(E,G)** cells following treatment with Globulol. Red fluorescence signifies the presence of EdU-positive cells, whereas blue fluorescence denotes Hoechst-positive cells. The data are expressed as the mean ± standard deviation (n = 3/group). NS means P > 0.05, *P < 0.05, **P < 0.01, ***P < 0.001 compared with the Ctrl group (0 μM Globulol).

The chemical structure of Globulol, sourced from the TCMSP database, is depicted in Figure 1A. The CCK8 assay revealed that Globulol significantly diminished the viability of both Huh1 and Huh7 cells in a dose-responsive manner. Treatment with Globulol for 24 h resulted in IC50 values of 136.6 μM and 141.5 μM for Huh1 and Huh7 cells, respectively, demonstrating statistically significant effects (*P* < 0.001, Figure 1B). Conversely, LX-2 cells (human hepatic stellate cell line) exposed to Globulol concentrations ranging from 30 to 160 μM exhibited no notable viability alterations (*P* > 0.05, Figure 1C). For subsequent experiments, dosages of 40 μM, 80 μM, and 120 μM were chosen. Furthermore, a marked decrease in EdU-positive Huh1 and Huh7 cells was observed following Globulol treatment, suggesting a suppression of cellular proliferation at these concentrations (*P* < 0.01, Figures 1D–G).

### 3.2 Globulol inhibits the cell cycle of HCC cells and induces apoptosis

**FIGURE 2:**
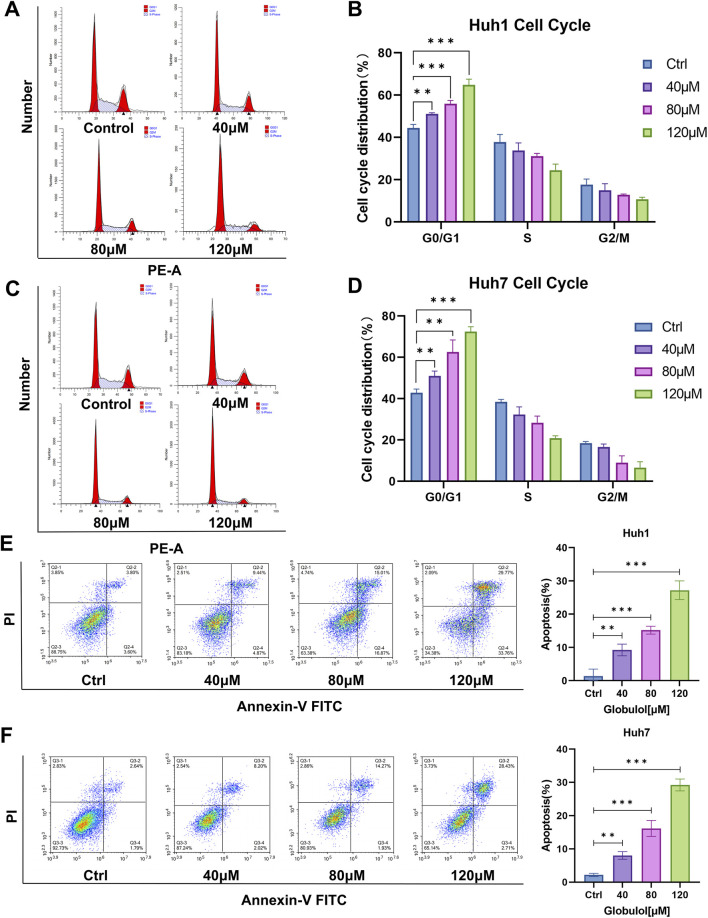
Effects of Globulol on cell cycle and apoptosis in HCC cells. Effects of Globulol on the cell cycle distribution of Huh1 **(A,B)** and Huh7 **(C,D)** cells. Globulol induced apoptosis in HCC cells **(E,F)**. The data are expressed as the mean ± standard deviation (n = 3/group). *P < 0.05, **P < 0.01, ***P < 0.001 compared with the Ctrl group (0 μM Globulol).

FC was employed to analyze cell-cycle distribution changes in Huh1 and Huh7 cells after treatment with 40 μM, 80 μM, and 120 μM Globulol for 24 h. Data from Figures 2A,B highlight a substantial increase in the G0/G1 phase fraction in Huh1 cells compared to controls (*P* < 0.001). Similarly, a significant elevation in the G0/G1 phase of Huh7 cells was observed post-treatment (Figures 2C,D) (*P* < 0.001). These findings suggest that Globulol effectively arrests cell cycle progression in these cell lines, thus inhibiting HCC cell proliferation. The impact of Globulol on apoptosis in Huh1 and Huh7 cells was also evaluated using FC. Figures 2E and F illustrate that varying concentrations of Globulol led to an increase in apoptosis rates, particularly at 40 μM (*P* < 0.01), 80 μM (*P* < 0.001) and 120 μM (*P* < 0.001). This dose-dependent induction of apoptosis suggests that Globulol effectively promotes cell death in these cell lines, further inhibiting HCC cell proliferation.

### 3.3 Globulol inhibits HCC tumor spheroid migration

**FIGURE 3:**
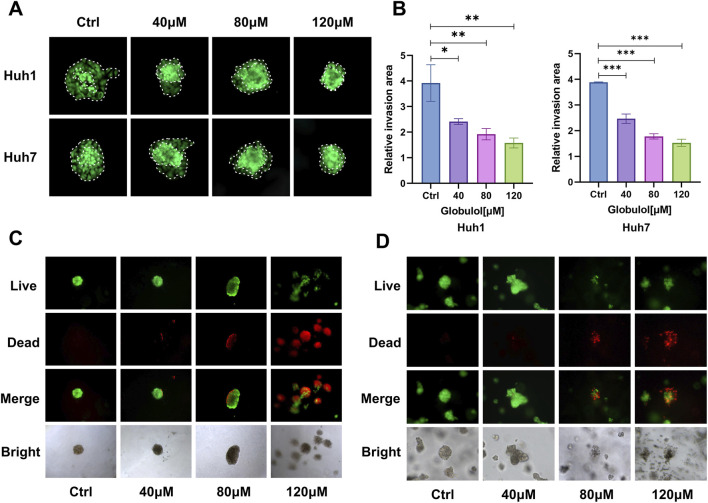
Effects of Globulol on tumor spheroid migration and structural integrity in HCC cell lines. The impact of Globulol on the migration of Huh1 **(A)** and Huh7 **(B)** cells. Live/dead staining results for the Huh1 **(C)** and Huh7 **(D)** cell lines, treated with Globulol for 24 h in a 3D tumor spheroid model, are presented. The data are expressed as the mean ± standard deviation (n = 3/group). *P < 0.05, **P < 0.01, ***P < 0.001 compared with the Ctrl group (0 μM Globulol).

3D tumor spheroids, which better mimic *in vivo* solid tumor architecture through tight cell-cell and cell-matrix interactions (Ishiguro et al., 2017), were used to assess the impact of Globulol on the migratory capacity of Huh1 and Huh7 cells. Figures 3A and B reveal that in the control group, tumor spheroids exhibited evident radial cell migration. Conversely, in groups treated with increasing concentrations of Globulol, the density of migrating cells decreased. At the highest concentration tested (120 μM), the boundary of the tumor spheroids was well-defined, with few migrating cells observed. These findings suggest that Globulol significantly suppresses the migratory capacity of both Huh1 (*P* < 0.01) and Huh7 cells (*P* < 0.001).

To further investigate Globulol’s penetration and cytotoxicity in a 3D model, tumor spheroids were used to simulate the drug-resistant TME. In the control group’s Huh1 and Huh7 models, tumor spheroids displayed uniform green fluorescence (live cells). However, in groups treated with Globulol, red fluorescence (dead cells) intensified significantly as the concentration increased. At the highest concentration (120 μM), the tumor spheroids showed signs of structural disintegration (Figures 3C,D). These results demonstrate Globulol’s ability to kill Huh1 and Huh7 cells within a 3D structure and disrupt tumor spheroid integrity.

### 3.4 Globulol involved in anti-tumor immune regulation

**FIGURE 4:**
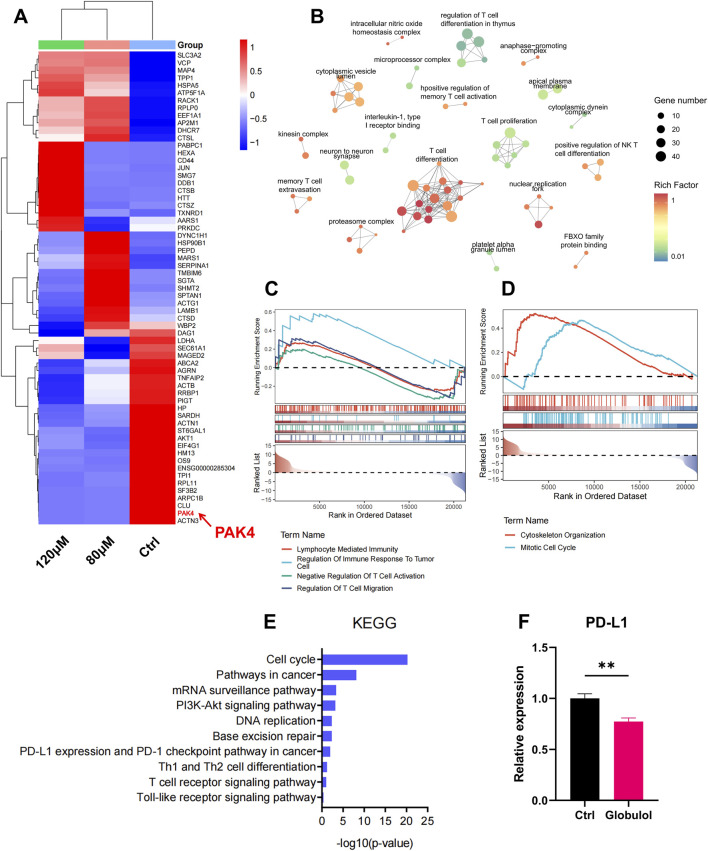
Globulol Involved in Anti-Tumor Immune Regulation. **(A)** Heatmap of differentially expressed genes: showing differentially expressed genes in Huh7 cells after Globulol treatment (red = upregulated, blue = downregulated), among which PAK4 was significantly downregulated; **(B)** GO functional enrichment analysis of all differentially expressed genes: enriched in immune-related pathways such as T cell proliferation/differentiation regulation; **(C,D)** Gene set enrichment analysis (GSEA): showing enrichment of pathways including lymphocyte-mediated immunity and T cell migration in the comparison of 120 μM Globulol vs. control (0 μM Globulol); **(E)** KEGG pathway enrichment analysis: focusing on cell cycle and PD-L1/PD-1 immune checkpoint pathways in the comparison of 120 μM Globulol vs. control (0 μM Globulol); **(F)** qRT-PCR verification: Globulol significantly reduced PD-L1 transcription level in Huh7 cells (P < 0.01). The data are expressed as the mean ± standard deviation (n = 3/group). **P < 0.01, compared with the Ctrl group (0 μM Globulol).

RNA-seq was employed to analyze gene expression changes in Globulol-treated Huh7 cells compared to untreated controls. Heat map were used to visualize these changes (Figure 4A). Additionally, GO analysis of all differentially expressed genes (Figure 4B) showed significant enrichment in pathways related to T cell proliferation and the regulation of T cell differentiation. Based on the initial 50 genes displayed in the heat map, p21-activated kinase 4 (PAK4) expression was significantly downregulated. The GSEA analysis (Figures 4C,D) revealed significant enrichment in pathways related to lymphocyte-mediated immunity, immune response regulation in tumor cells, T cell migration, cytoskeleton organization, and the mitotic cell cycle. KEGG analysis (Figure 4E) indicated significant enrichment in the cell cycle pathway, PD-L1 expression, and the PD-1 immune checkpoint pathway. qRT-PCR was used to validate the differential gene expression in Huh7 cells following Globulol treatment (Figure 4F), which demonstrated a significant reduction in PD-L1 transcription (*P* < 0.01). Given that PAK4 is overexpressed in various tumor tissues and is linked to malignancy and poor prognosis in HCC, it was selected for further investigation.

### 3.5 Globulol-treated HCC cells promote the migration of CD8^+^T Cells

**FIGURE 5:**
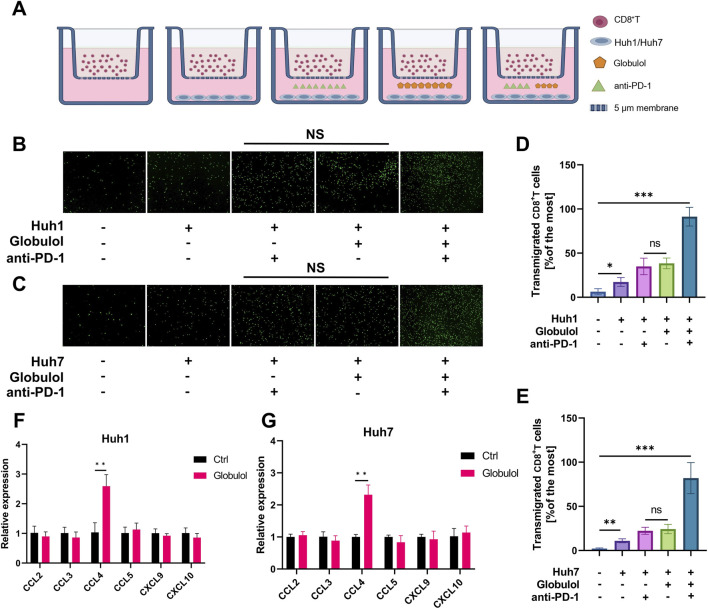
Globulol enhanced recruitment of CD8^+^T cells. **(A)** Assessment of the migratory behavior of human CD8^+^T cells in response to HCC cell lines following exposure to Globulol or TIS. The chemotactic response of activated CD8^+^T cells towards Huh1 **(B)** and Huh7 **(C)** HCC cells. Measurement of the migratory rate of Huh1 **(D)** or Huh7 **(E)** cells in response to activated CD8^+^T cells. qRT-PCR analysis was conducted to assess the expression levels of chemokines in Huh1 **(F)** or Huh7 **(G)** cells following treatment with Globulol or TIS. The data are expressed as the mean ± standard deviation (n = 3/group). ns means P > 0.05, *P < 0.05, **P < 0.01, ***P < 0.001 compared with the Ctrl group (0 μM Globulol).

Transwell coculture assays were performed to investigate the influence of Globulol on CD8^+^T-cell chemotaxis towards HCC cell lines treated with either Globulol or the anti-PD-1 drug TIS, as depicted in Figure 5A. Enhanced migration of CD8^+^T cells was observed in both Huh1 and Huh7 cells post-treatment (Figures 5B–E), respectively. Further augmentation of CD8^+^T-cell migration was noted in the combination group with TIS. To identify potential chemokines involved in this process, qRT-PCR was performed to detect the expression levels of multiple chemokines in Globulol- or TIS-treated Huh1 and Huh7 cells. Among the detected chemokines, only CCL4 showed a significant upregulation in both Huh1 (Figure 5F) and Huh7 cells (Figure 5G) after Globulol treatment, while the expression of other chemokines remained unchanged.

### 3.6 Globulol enhances CD8^+^T Cell proliferation and cytotoxicity, and enhances the pharmacological effects of anti-PD-1 drugs in combination

**FIGURE 6:**
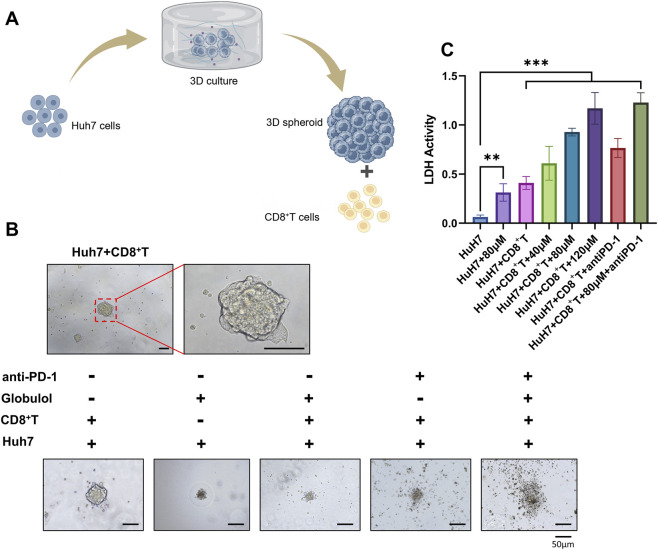
The effect of Huh7 cells treated with Globulol or TIS on CD8^+^T cell function. **(A)** Schematic diagram of co-culture of Huh7 3D tumor spheroids with human CD8^+^T cells. **(B)** Co-culture results of Huh7 3D tumor spheroids treated with Globulol (80 μM) or TIS(57.14 mg/L) with CD8^+^T cells. **(C)** LDH levels in the medium after co-culture with different concentrations of Globulol combined with TIS(57.14 mg/L). The data are expressed as the mean ± standard deviation (n = 3/group). ***P < 0.001 compared with the Huh7 group (0 μM Globulol).

To assess the combination effect of Globulol combined with TIS, an *in vitro* 3D tumor spheroid co-culture model of Huh7 cells and CD8^+^T cells was established (Figure 6A). Figure 6B reveals that in the control group, the tumor spheroids maintained intact structures with clear edges and no significant cell detachment. Treatment with Globulol resulted in slight loosening of the spheroid edges and some cell detachment. Following TIS treatment, the spheroid structure remained largely intact with localized disintegration. However, after combined treatment with Globulol and TIS, significant disintegration occurred, the core area collapsed, and numerous fragmented cells detached. These observations indicate that the combination of Globulol and TIS exerts a combination effect, which significantly outperforms either monotherapy alone. LDH assay results (Figure 6C) demonstrated that Globulol enhanced the cytotoxicity of CD8^+^T cells in a concentration-dependent manner, and this pharmacological effect was further potentiated when Globulol was combined with TIS (*P* < 0.001).

**FIGURE 7:**
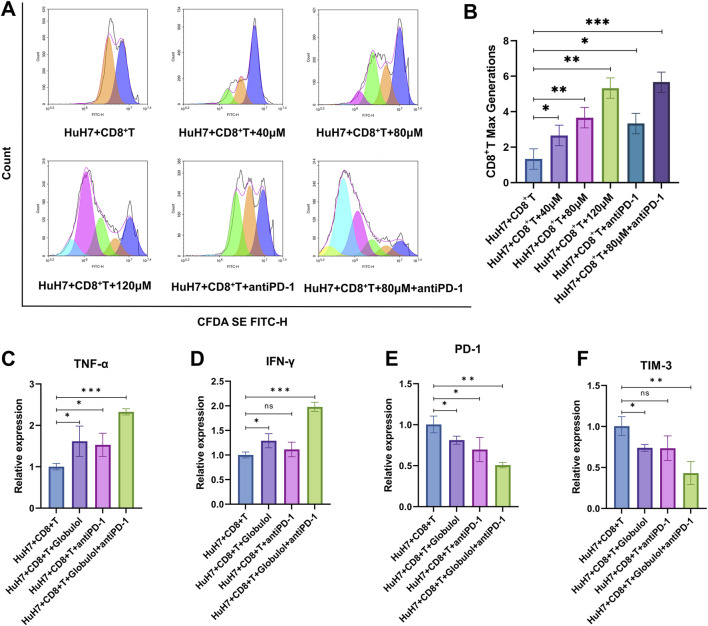
Globulol promotes the proliferation of CD8+T cells and enhances the pharmacological effects of Anti-PD-1 Drugs in combination. **(A,B)** FC observed the proliferation levels of CD8^+^T cells following treatment with varying concentrations of Globulol in conjunction with TIS. **(C–F)** qRT-PCR Analysis of TNF-α, IFN-γ, PD-1, and TIM-3 expression levels in CD8^+^T cells co-cultured with Huh7 cells treated with Globulol or TIS. The data are expressed as the mean ± standard deviation (n = 3/group). ns means P > 0.05, *P < 0.05, **P < 0.01, ***P < 0.001 compared with the Huh7+ CD8^+^ T group (0 μM Globulol).

The influence of Globulol on the proliferation of CD8^+^T cells was investigated using FC. Results presented in Figure 7A and B demonstrate that Globulol facilitated a dose-dependent increase in CD8^+^T cell proliferation, which was further potentiated when combined with TIS (*P* < 0.001). This combined treatment also markedly increased the expression levels of TNF-α and IFN-γ (*P* < 0.001) (Figures 7C,D), while reducing the expression of PD-1 and TIM-3 (*P* < 0.01) (Figures 7E,F). Notably, CD8^+^T cells from the combination treatment group showed elevated levels of TNF-α and IFN-γ compared to those from the untreated group.

### 3.7 Globulol inhibits PD-L1 expression via the PAK4-pAKT-STAT3 pathway

**FIGURE 8:**
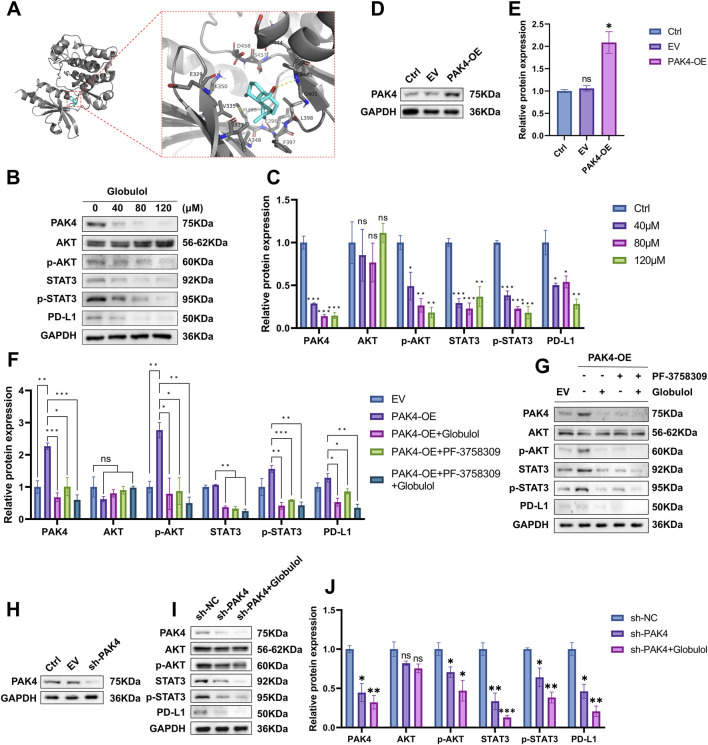
Globulol inhibits PD-L1 expression through the PAK4-pAKT-STAT3 pathway. **(A)** Binding mode diagram of Globulol and PAK4 ligand. **(B,C)** Impact of various Globulol concentrations on the target protein expression. **(D,E)** Protein expression levels in PAK4-overexpressing cells. **(F,G)** Effects of combined treatment with Globulol and a PAK4 inhibitor (PF-3758309) on target protein expression in PAK4-overexpressing cells. **(H)** Protein levels after PAK4 knockdown. **(I,J)** Impact of Globulol treatment on protein expression in PAK4-knocked-down cells. The data are expressed as the mean ± standard deviation (n = 3/group). ns means P > 0.05, *P < 0.05, **P < 0.01, ***P < 0.001, EV means empty vector group, Ctrl group (0 μM Globulol).

RNA-seq differential gene analysis identified PAK4 as the primary target. Figure 8A illustrates the binding mode of Globulol with PAK4 (binding energy: −6.8 kcal/mol). PAK4 is shown as a gray ribbon, and Globulol fits snugly into PAK4’s binding pocket. The complex is stabilized by a hydrogen bond with residue A402, hydrophobic contacts (G401, F397, L398), and polar interactions (D458, D444, E396, K350). These favorable binding energy and stable interactions support Globulol’s potential as a PAK4-targeted agent. PAK4 regulates the WNT/β-catenin and promotes PI3K/AKT signaling by directly binding to PI3K, thus enhancing AKT phosphorylation (Naїja et al., 2021). This activation augments STAT3-dependent PD-L1 expression downstream (Zhang et al., 2018). Western blot analysis was conducted to measure key protein expressions in Huh7 cells treated with varying Globulol concentrations. Figure 8B and C show that Globulol significantly reduced the protein levels of PAK4, p-AKT (Ser473), STAT3, p-STAT3 (Y705), and PD-L1, without notably affecting total AKT levels (compared with Ctrl group). Additionally, a Huh7 cell model with PAK4 overexpression (PAK4-OE) was established (Figures 8D,E). This model, treated with a combination of Globulol and the PAK4 inhibitor PF-3758309, demonstrated that PAK4 overexpression increased p-AKT (Ser473) protein levels but did not affect total AKT levels (*P* > 0.05). Treatment with Globulol significantly decreased the protein expressions of PAK4, p-AKT (Ser473), STAT3, p-STAT3 (Y705), and PD-L1. Further combination with the PAK4 inhibitor resulted in additional reductions in these proteins (compared with the PAK4-OE group) (Figures 8F,G). Similarly, silencing PAK4 (sh-PAK4) markedly reduced these protein levels, and additional treatment with Globulol further decreased the levels of PAK4, STAT3, p-STAT3 (Y705), and PD-L1 (Figures 8H–J). These findings confirm that Globulol can effectively inhibit PD-L1 expression and improve the immunosuppressive microenvironment by modulating the PAK4-pAKT-STAT3 pathway.

### 3.8 High expression of PAK4 in HCC negatively impacts clinical prognosis

**FIGURE 9:**
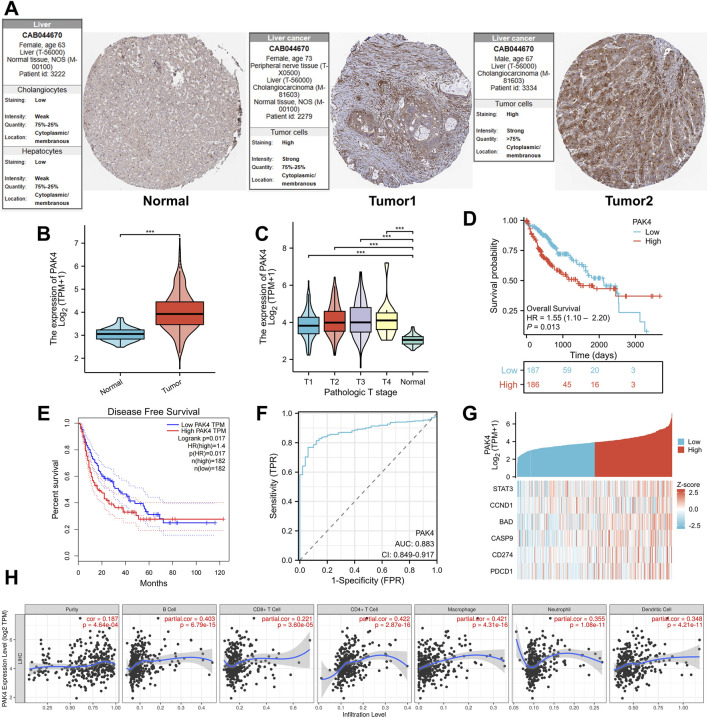
High expression of PAK4 in HCC negatively impacts clinical prognosis. **(A)** IHC analysis of PAK4 gene expression in clinical samples from the HPA database. **(B)** Differential expression of the PAK4 gene between cancerous and normal tissues. **(C)** PAK4 expression across different pathological stages of HCC. **(D)** Relationship between PAK4 expression levels and overall survival rate. **(E)** Impact of PAK4 expression on disease-free survival. **(F)** ROC curve for the PAK4 gene. **(G)** Correlation between PAK4 and downstream gene expression. **(H)** Analysis of the correlation between PAK4 and immune cell infiltration levels.

Immunohistochemistry analysis was employed to evaluate the expression of PAK4 protein in both normal and cancerous tissues. The findings revealed a notable upregulation of PAK4 in cancerous tissues (Figures 9A,B). The expression of PAK4 also varies across different cancer types and different pathological stages of HCC (Figure 9C). Notably, elevated PAK4 levels were significantly correlated with reduced overall survival and disease-free survival rates (Figures 9D,E). An AUC value close to 1.0 suggests a reliable detection method, with the AUC for PAK4 being 0.883, indicating its reliability as a clinical diagnostic marker for HCC (Figure 9F). The expression level of the PAK4 gene was positively correlated with genes such as STAT3, CD274 (PD-L1), and PDCD1 (PD-1) (Figure 9G). The TIMER database was used to explore the relationship between PAK4, PDCD1 (PD-1) gene expression levels and the infiltration of six types of immune cells in LC tissues, showing significant correlations (Figure 9H).

## 4 Discussion

HCC is increasingly common globally, with a prognosis that remains poor. Its rising incidence is strongly linked to various factors, including chronic viral hepatitis (HBV, HCV), alcoholic liver disease, and metabolic syndrome-related fatty liver disease (Singal et al., 2020). Traditional Chinese medicine (TCM) demonstrates advantages in tumor treatment through its multi-metabolite synergy and multi-target intervention. Recently, TCM monomers, the key metabolite underlying TCM’s pharmacological effects, have emerged prominently in anti-tumor research (Li et al., 2021). Immune checkpoint inhibitors (ICIs), particularly anti-PD-1 drugs, have made progress in treating HCC. However, the pharmacological effects of monotherapy is limited by issues such as safety concerns, and primary or secondary drug resistance. Therefore, developing new drugs to enhance the pharmacological effects of ICIs holds significant clinical importance.

Terpenoid metabolites were generally considered as the major bioactive metabolites in *Alpinia oxyphylla* Miq. Their antitumor pharmacological properties have gained significant recognition, and terpenoid-based drugs such as β-elemene and ganoderma triterpenes are now used clinically (Guo et al., 2024). Globulol, extracted from *Alpinia oxyphylla* Miq., has shown anticancer potential. In our study, Globulol was found to inhibit proliferation and migration of Huh1 and Huh7 cells, induce G0/G1 phase arrest and apoptosis, and selectively spare LX-2 cells (Figures 1–3). Similarly, Globulol augments the cytotoxicity of CD8^+^T cells in a concentration-dependent manner and stimulates the proliferation of CD8^+^T cells (Figures 6, 7A,B).

Cytotoxic T lymphocytes, or CD8^+^T cells, play a pivotal role in targeting tumors (Peña-Asensio et al., 2021). The TME of HCC is often characterized by diminished lymphocyte infiltration (Chen et al., 2023). Increased CD8^+^T cell infiltration correlates with longer recurrence-free survival and decreased recurrence rates in HCC (Ju et al., 2021). Chemokines within the TME recruit immune cells, influencing tumor progression by either suppressing or enhancing immunity (Del Prete et al., 2017). This study assessed the impact of Globulol on CD8^+^ T cell infiltration by analyzing chemokine levels. Our results demonstrate that Globulol, combined with TIS,enhancing CD8^+^T cell chemotaxis and migration (Figure 5), thus improving the tumor immune environment. PD-1, primarily expressed on activated T and NK cells (Huang et al., 2021), when bound by overexpressed PD-L1/2 from tumor cells, triggers immune-suppressive pathways facilitating immune evasion (Brahmer et al., 2012). Inhibitors of PD-1/PD-L1 interactions restore immune functionality and effectively eradicate tumor cells (Jiang et al., 2019). TIS, a PD-1 inhibitor, is approved as a primary treatment for unresectable or metastatic HCC (Yi et al., 2022). Our findings indicate that Globulol reduces PD-L1 expression in HCC cells (Figure 8), when used in conjunction with TIS, increases CD8^+^T cell activation and cytotoxicity, thereby enhancing TIS’s pharmacological effects (Figures 7C–F).

PAK4, a serine/threonine kinase (Wu and Jiang, 2022), is highly expressed in various digestive system tumors, particularly in metastatic versus primary HCC tissues. Its overexpression correlates with malignancy and adverse outcomes in HCC (Lu et al., 2017). Research has demonstrated increased T cell and NK cell infiltration in PAK4-knockout mouse tumor tissues, and resistance to anti-PD-1 medications is reversed (Abril-Rodriguez et al., 2019). Mice treated with both anti-PD-1 drugs and a PAK4 inhibitor (KPT-9274) show superior anti-tumor responses compared to those treated solely with anti-PD-1 drugs (Ma et al., 2020). Treatment of pancreatic cancer cells with a PAK1/4 inhibitor (PF-3758309) has also been shown to downregulate PD-L1 expression and increase lymphocyte-induced tumor cell death (Wang et al., 2020). PAK4 enhances PI3K/AKT signaling in various cancers by directly interacting with PI3K, promoting AKT phosphorylation (Cheng et al., 2022). Activation of this pathway increases PD-L1 expression across different tumors (He et al., 2021). STAT3, vital for tumor immune evasion, regulates PD-L1 expression, influencing the immunosuppressive TME (Waldmann and Chen, 2017). A recent study has found that activation of the PI3K/AKT pathway augments STAT3-dependent PD-L1 expression in tumors. Knocking down STAT3 leads to reduced PD-L1 expression in melanoma cells (Jiang et al., 2013). Our results indicate that Globulol specifically targets and suppresses PAK4 expression in HCC. This inhibition leads to reduced expression of downstream pAKT, STAT3, pSTAT3, and PD-L1, while the total AKT level remains constant (Figure 8), suggesting that PAK4’s influence on downstream AKT primarily affects kinase activity rather than the synthesis or degradation of the protein.

Our findings underscore the potential clinical significance of combining PD-1 blockade with Globulol. This study is solely based on *in vitro* cell cultures and 3D tumor spheroid models; we have not yet detected its effects on the activity and dose dependence of SHP1/SHP2—key molecules in T cell responses to PD-L1—and thus, the *in vivo* antitumor activity of Globulol and its combination effects with TIS have not been validated using the mouse orthotopic xenograft model of HCC. Accordingly, future studies should integrate mouse orthotopic liver xenograft models with primary LC models to further elaborate on, validate these findings, and improve the elucidation of the molecular mechanism by which Globulol regulates anti-tumor immunity. It should be noted as a limitation that while we detected significant CCL4 upregulation in HCC cells and increased CD8^+^T cell recruitment following Globulol treatment in the co-culture system, functional validation was not performed to confirm their direct causal relationship, which requires further investigation. Furthermore, the pharmacokinetics of Globulol must be thoroughly characterized in forthcoming clinical trials, which could significantly advance its potential as an anticancer immunotherapy agent.

## 5 Conclusion

Our study show that Globulol, derived from the traditional Chinese medicinal botanical drug *Alpinia oxyphylla* Miq., exerts anti-HCC effects: it concentration-dependently inhibits Huh1/Huh7 cell viability, proliferation, and migration while sparing LX-2 cells. Critically, it targets the PAK4-pAKT-STAT3 axis to downregulate HCC PD-L1, enhances CD8^+^T cell infiltration, and exerts combined pharmacological effects with anti-PD-1 drug Tislelizumab (TIS)—boosting CD8^+^ T cell proliferation/cytotoxicity, lowering T cell PD-1/TIM-3, and improving TIS pharmacological effects. These findings support Globulol + anti-PD-1 as a promising HCC therapy; future work will validate *in vivo* via HCC xenografts and analyze Globulol pharmacokinetics to advance translation.

## Data Availability

The data presented in the study are deposited in the NCBI BioProject repository, BioProject accession number PRJNA1420507.
